# Supplementary material for: Ni-Catalyzed Enantioselective Intramolecular Mizoroki–Heck Reaction for the Synthesis of Phenanthridinone Derivatives
Source: J Org Chem. 2023 Jun 15;88(13):8203–26. doi: 10.1021/acs.joc.3c00202 (PMC10337041; doi:10.1021/acs.joc.3c00202)
Supplement: Supplementary file 2 — jo3c00202_si_002.zip [file jo3c00202_si_002.zip › Heck products/2g CH3CO2CH2 NMe Heck/1H CH3CO2CH2 Heck 2g/1/pdata/1/email_wm 104-158 fr 7_1_1.pdf]

wm 104-158 fr 7
PROTON CDCl3 {D:\nmrusers\malachowski} BM 9

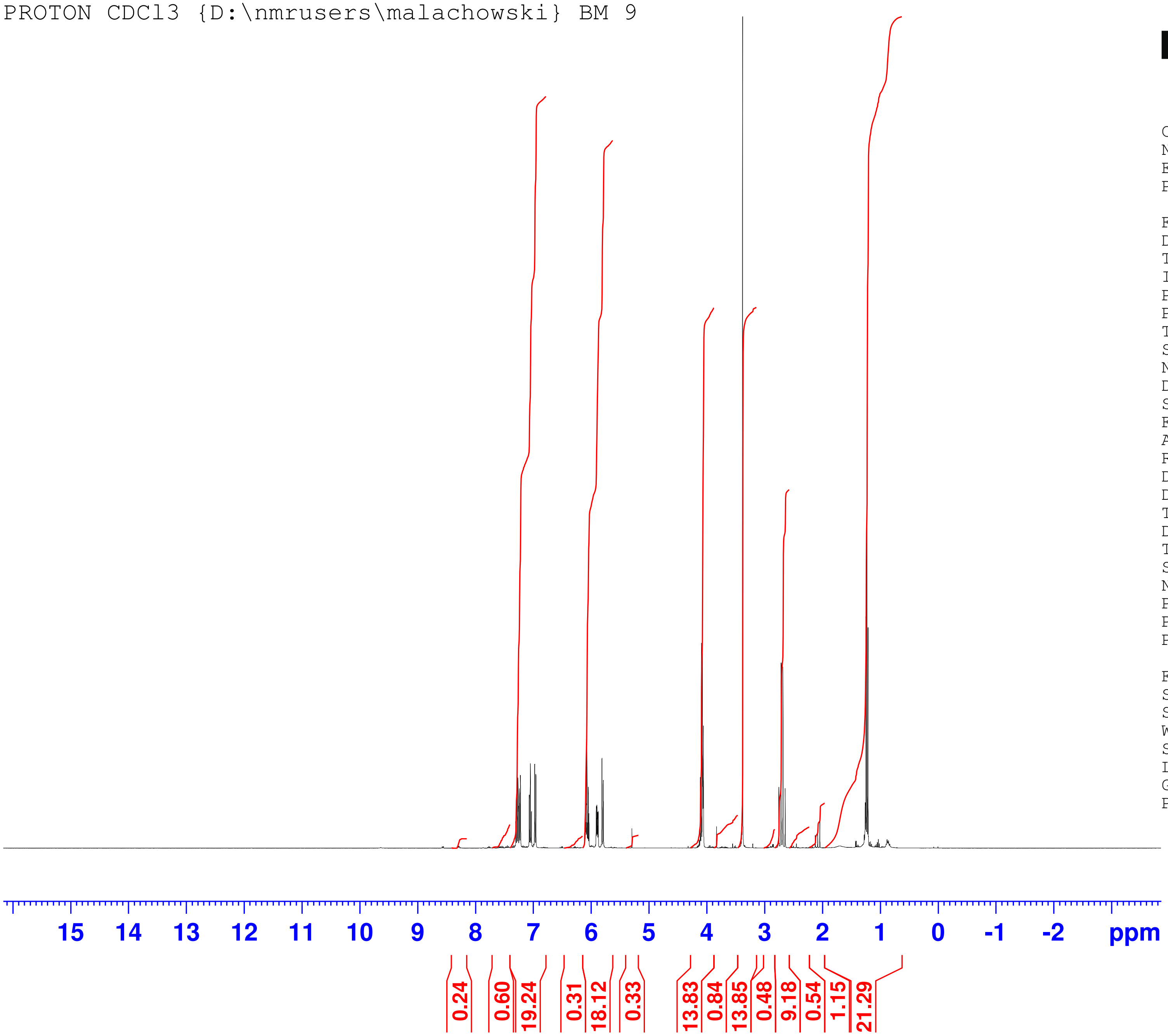

BRUKER

Current Data Parameters

NAME	wm 104-158 fr 7
EXPNO	1
PROCNO	1

F2 - Acquisition Parameters

Date_	20210216
Time	17.20 h
INSTRUM	spect
PROBHD	Z104450_0352 (
PULPROG	zg30
TD	65536
SOLVENT	CDCl3
NS	16
DS	2
SWH	8012.820 Hz
FIDRES	0.244532 Hz
AQ	4.0894465 sec
RG	128
DW	62.400 usec
DE	17.30 usec
TE	298.0 K
D1	1.00000000 sec
TD0	1
SFO1	400.1524709 MHz
NUC1	1H
P0	4.25 usec
P1	12.75 usec
PLW1	12.14200020 W

F2 - Processing parameters

SI	65536
SF	400.1500081 MHz
WDW	EM
SSB	0
LB	0.30 Hz
GB	0
PC	1.00
